# Structure of noncoding RNA is a determinant of function of RNA binding proteins in transcriptional regulation

**DOI:** 10.1186/2045-3701-2-1

**Published:** 2012-01-03

**Authors:** Takanori Oyoshi, Riki Kurokawa

**Affiliations:** 1 Department of Chemistry, Faculty of Science, Graduate School of Science, Shizuoka University, 836 Oya, Suruga, Shizuoka 422-8529, Japan; 2 Division of Gene Structure and Function Research Center for Genomic Medicine Saitama Medical University, 1397-1 Yamane, Hidaka-shi, Saitama-Ken, Japan, Mail code 350-1241

**Keywords:** noncoding RNA, EWS, TLS, B2 RNA, G-quadruplex, TERRA

## Abstract

The majority of the noncoding regions of mammalian genomes have been found to be transcribed to generate noncoding RNAs (ncRNAs), resulting in intense interest in their biological roles. During the past decade, numerous ncRNAs and aptamers have been identified as regulators of transcription. 6S RNA, first described as a ncRNA in *E. coli*, mimics an open promoter structure, which has a large bulge with two hairpin/stalk structures that regulate transcription through interactions with RNA polymerase. B2 RNA, which has stem-loops and unstructured single-stranded regions, represses transcription of mRNA in response to various stresses, including heat shock in mouse cells. The interaction of TLS (translocated in liposarcoma) with CBP/p300 was induced by ncRNAs that bind to TLS, and this in turn results in inhibition of CBP/p300 histone acetyltransferase (HAT) activity in human cells. Transcription regulator EWS (Ewing's sarcoma), which is highly related to TLS, and TLS specifically bind to G-quadruplex structures *in vitro*. The carboxy terminus containing the Arg-Gly-Gly (RGG) repeat domains in these proteins are necessary for *cis*-repression of transcription activation and HAT activity by the N-terminal glutamine-rich domain. Especially, the RGG domain in the carboxy terminus of EWS is important for the G-quadruplex specific binding. Together, these data suggest that functions of EWS and TLS are modulated by specific structures of ncRNAs.

## Introduction

Gene silencing has emerged as one of the major functions of short double stranded noncoding RNAs (ncRNAs) that are generated by specific processing machinery. The mechanisms by which small ncRNAs, siRNAs and miRNAs, participate in RNAi pathway involved in gene silencing, mRNA stability and translation arrest have been extensively studied [1,2]. In contrast, regulatory functions of other classes of ncRNAs are much less well understood. Transcription is also regulated by other classes of ncRNAs, including long, single-stranded, polyadenylated RNA molecules. Recently, ncRNAs and synthetic RNA oligonucleotides (RNA aptamers) have been found to exert inhibitory effects on transcription through inhibition of histone acetyltransferase (HAT). The inhibitory effect was achieved through blocking function of transcription machinery with conformational changes. In this review, we describe inhibitory mechanisms used by divergent ncRNAs and discuss common structures of these ncRNAs involved in regulation of transcription. Recently, a guanine-rich structure has been found to exert regulatory roles in eukaryotic transcription. Therefore, we also focus on regulatory functions of the guanine-rich structure in transcription.

### 6S RNA inhibits RNA polymerase II in *E. coli*

6S RNA was first describe as a ncRNA in *E. coli *[3]. 6S RNA mimics an open promoter structure and regulates transcription through interaction with RNA polymerase in bacterial cells [4]. The structure of 6S RNA shows a large bulge of two single strands between the stalk and the hairpin structures. Bacterial RNA polymerase is a multi-subunit enzyme consisting of a core enzyme and a specific subunit, forming the holoenzyme [5]. The 6S RNA sequence surrounding the bubble has contacts directly with both the σ^70 ^and β/β' polymerase subunits in the holoenzyme [6-8]. 6S RNA accumulates as cells reach the stationary phase of growth and mediates phase-specific change of RNA polymerase [4,9]. 6S RNA represses expression from a σ^70^-dependent promoter during the stationary phase [4]. The binding of 6S RNA with RNA polymerase modulates the σ^70^-holoenzyme activity. The binding of the 6S RNA competes with binding of the RNA polymerase to the promoter regions. The bacterial RNA polymerase utilizes the 6S RNA as a template and generates short (14- to 20-nt) RNA products that are initiated within the bubble [6,10]. The RNA products form a triplex-helix hybrid with the 6S RNA hairpin. This hybrid might destabilize the RNA polymerase-6S RNA complex, and rescue polymerase activity from the repressed status.

Reducing the size of the single-stranded region of 6S RNA with deletion mutation destroys activity. The alteration of sequences to induce base-paring throughout the region of 6S RNA also results in producing an inactive RNA, suggesting that the structure is crucial [8]. However, the enlargement of the overall size of the single-stranded DNA at the bulge region of 6S RNA had no effect on binding to RNA polymerase, indicating that there are not precise size requirements for the bulge region [8].

### B2 RNA represses transcription by RNA polymerase II in mouse cells

B2 RNA is likely to be a eukaryotic counterpart of the bacterial 6S RNA and a small ncRNA of 178 nt transcribed by RNA polymerase III from short interspersed elements (SINEs). Expression of B2 RNA was increased in response to transformation by simian virus 40 and various stresses, including UV exposure, gamma radiation, and heat shock in mouse cell [11-20]. B2 RNA proposed to contribute to the repression of the transcription of house-keeping genes like actin and hexokinase II after treatment with heat-shock stress [14].

B2 RNA binds RNA polymerase and inhibits transcription [21]. Through this binding to RNA polymerase II, B2 RNA is incorporated into preinitiation complexes at promoters and inhibits transcription. The 70 nucleotides (nts) at the 5' end of B2 RNAs are evolutionarily conserved with tRNAs, and the 3' ends of B2 RNAs contain an A-rich sequence also conserved among all short interspersed elements, which are retrotransposons dispersed throughout the mouse genome with ~350,000 copies per cell [22]. Biochemical assay shows that the 5' region from 3 to 74 nts neither binds to polymerase nor represses transcription, whereas the region from 75 to 149 binds to RNA polymerase II and represses transcription [23]. Further deletion experiments revealed that the region from 81 to 131, which contains a stem-loop (81-97) and the unstructured single-stranded region (98-115), can be assembled into the preinitiation complexes and also represses transcription.

### ncRNAs and G-rich RNA binding to TLS

It has been published that ncRNAs transcribed from the promoter regions of cyclin D1 gene (promoter-associated ncRNAs) have regulatory roles in transcription [24]. Evidence that the promoter-associated ncRNAs execute transcriptional regulation, has been recently provided by analysis of TLS (translocated in liposarcoma) [24]. TLS was initially identified as TLS-CHOP, the fusion protein arising from a chromosomal translocation [25,26]. TLS also has been found to be involved in numerous activities, including transcription control, mRNA processing and DNA repair [27-29].

Biochemical studies demonstrated that TLS could bind to CBP/p300, which serve as essential coactivator for divergent species of transcription factors, for example, CREB, nuclear receptors, NFκB and STAT transcription factors [30,31]. Their function of coactivator is mainly generated by their intrinsic histone acetyltransferase (HAT) activity [32,33]. Furthermore, TLS could strongly inhibit the CBP/p300 HAT activity on the core histones and other substrates [24].

**Figure 1:**
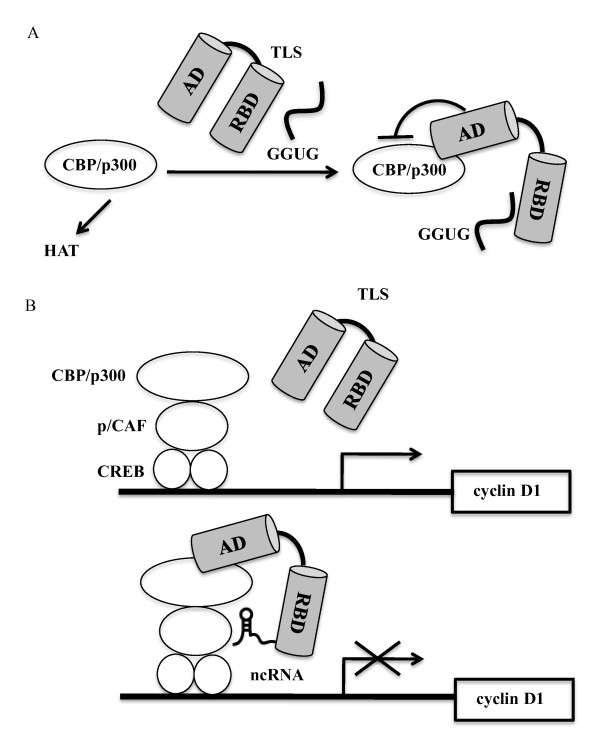
**Mechanism of RNA-dependent histone acetyltransferase (HAT) activities regulated by TLS**. (A) HAT regulation by TLS and GGUG. (B) Transcriptional regulation by TLS and ncRNAs. AD: activation domain, RBD: RNA binding domain, p/CAF: p300/CBP-associated factor.

It has been reported that synthetic RNA oligonucleotides bind to TLS through a consensus sequence GGUG [34]. Upon binding of GGUG RNA oligonucleotides (GGUG RNA), inhibitory activity of TLS against the HAT activity of CBP/p300 was enhanced [24]. Biochemical experiments showed that the carboxy terminus of TLS bound to GGUG RNA, whereas the N terminus was interacted with CBP. The N terminus of TLS alone has stronger inhibitory activity against the HAT than did the full-length TLS. Furthermore, the N terminus of TLS turned out to interact with the C terminus of TLS (Figure 1A). The lower inhibitory activity of the full-length TLS might be the outcome of blocking the binding of the N-terminal domain of TLS with the HAT domain of CBP/p300 through binding of the TLS C-terminus. TLS bound with GGUG RNAs induced its higher binding to CBP/p300. These data suggest that the stronger inhibition of the HAT activity by TLS bound GGUG RNA results from bound of the C-terminus of TLS with GGUG RNA (Figure 1A) [34].

The interaction of TLS with CBP/p300 was stimulated by RNA oligonucleotides that binds to TLS, and this in turn resulted in inhibition of CBP/p300 HAT activity (Figure 1B) [24]. Cell-based studies demonstrated that TLS could be recruited to and repress a subset of CREB target genes, including cyclin D1 and cyclin E1, by reducing local histone acetylation. Intriguingly, recruitment of TLS appeared to be dependent on its binding to ncRNAs transcribed from promoter regions of the cyclin D1 gene. Furthermore, transcription of these promoter-associated ncRNAs was induced by ionizing radiation, enhancing TLS recruitment and reducing cyclin D1 expression. Taken together, these findings provide the basis for a model in which the promoter-associated ncRNAs recruit TLS to the cyclin D1 promoter [24]. The interaction of these ncRNAs with TLS induces recruitment of TLS to the promoter region of cyclin D1, and also a conformational change of TLS. This conformational change of TLS enables it to interact with CBP/p300 and to inhibit their HAT activities.

**Figure 2:**
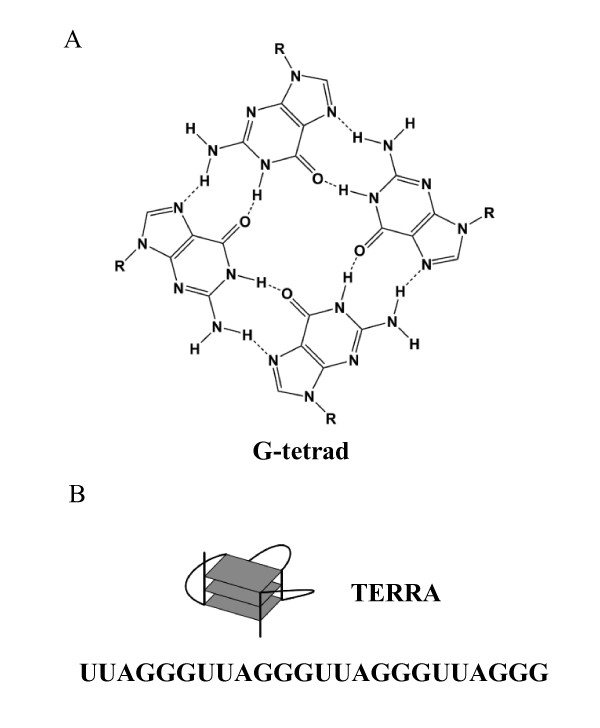
**Structural features of human telomeric repeat containing RNA (TERRA) formed into G-quadruplex**. (A) Structure of G-tetrad consisting of G-quadruplex. (B) G-quadruplex structure and sequences of TERRA.

RNA bearing Guanine-rich sequences is able to fold into a G-quadruplex structure with cyclic Hoogsteen base pairs of four guanine bases (G-tetrads; Figure 2A) [35,36]. The formation of the G-quadruplex is stabilized by the presence of monovalent cations (e.g., Na^+^, K^+^), which are positioned in the center of the structure and coordinated by the electron-rich carbonyl oxygens. The NMR structure of human telomeric repeat containing RNA (TERRA) r(UUAGGG)_4 _with K^+ ^has been solved as the G-quadruplex structure *in vitro *and the structure of TERRA in living cells has been confirmed as G-quadruplex by a lights-switching pyrene probe (Figure 2B) [37].

TERRA is a large non-coding RNA in fungi and animals and works as a direct inhibitor of human telomerase, forming an integrated component of telomeric heterochromatin in cellular nuclei [38-41]. TLS strongly binds to TERRA via G-quadruplex structure *in vitro *[42]. Purification of the human telomeric chromatin using proteomics technology indicated that TLS is one component of telomere binding protein goups [43]. TLS forms a complex with the heterogeneous nuclear ribonucleoprotein A1 (hnRNP A1) which contains the four RNA recognition motifs and the RGG domain in the C-terminal domain and binds to TERRA and human telomeric DNA [44-46]. hnRNP A1 modulates telomere length and displaces the replication protein A (RPA) for protection of binding of telomeres 1 (POT 1) to telomeric DNA.

### TERRA, G-quadruplex RNA, binds to transcriptional factor EWS

**Figure 3:**
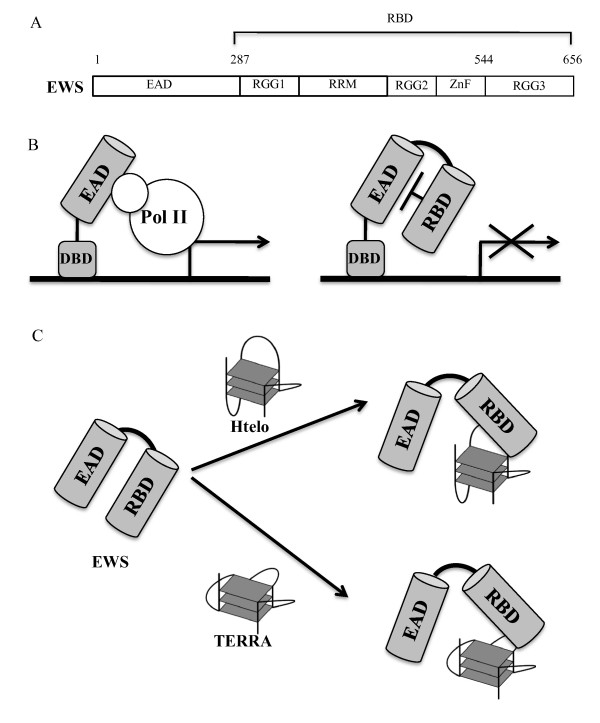
**Transcriptional features and the G-quadruplex binding model of EWS**. (A) Structural features of EWS. EAD: EWS activation domain, RGG: Arg-Gly-Gly domain, RRM: RNA recognition motif, Znf: zinc finger. (B) Mechanism of RNA binding domain-dependent transcriptional regulation of EWS. DBD: GAL4 DNA binding domain. (C) The G-quadruplex binding model by EWS RBD containing RGG domain.

EWS is homologous to TLS and TAF15. These three proteins form the TET family (Figure 3). The current knowledge of EWS (Ewing's sarcoma) has been provided mainly from analysis of dominant oncogenes that arise due to chromosomal translocations in which EWS is fused to a variety of cellular transcription factors [28,47,48]. The EWS fusion proteins are potent transcription activators that require the EWS N-terminal domain and the C-terminal DNA-binding domain contributed by fusion partners [49-54]. For instance, a fusion gene EWS-ATF1 is a potent constitutive activator of ATF-dependent promoters [55]. The N-terminus of EWS binds directly to a subunit (hsRPB7) of RNA polymerase II and the interaction is thought to be important for transactivation [56].

The N-terminal regions of the TET proteins contain the glutamine-rich domain that is a common structure of transcription activation domain, while the C-terminal half of the TET proteins contains a series of RNA binding regions, including an RNA recognition motif (RRM), a C_2_C_2 _zinc finger (ZnF) and three Arg-Gly-Gly (RGG) repeat domains (Figure 3A) [57,58]. The RGG domain within the carboxy terminus of EWS is necessary for *cis*-repression of transcription induced by the N-terminus of the glutamine-rich domain (Figure 3B) [58,59]. The transcription repression might result from the carboxy-terminal RGG domain that blocks interaction between the N-terminus of the glutamine-rich domain and hsRPB7.

Similar to TLS, EWS could bind to TERRA through the G-quadruplex in a structure-specific manner (Figure 3C) [60,61]. Binding experiments showed that the carboxy-terminal RGG domain of EWS specifically bound to G-quadruplex RNA, whereas the proteins containing the N-terminal of the glutamine-rich domain, RRM, ZnF and other RGG domains did not [60]. The carboxy-terminal RGG domain of EWS contributes not only to the binding to G-quadruplex RNA but also that to G-quadruplex DNA binding. EWS binds preferentially to G-quadruplexes with the longer loops. The electrophoresis mobility shift assay using EWS with G-quadruplexes containing abasic sites instead of nucleotides in the loops indicates that the carboxy-terminal RGG domain of EWS recognizes the phosphate backbone of the loops in G-quadruplexes. The findings could contribute to analyzing the nucleic acids binding protein that selectively target the G-quadruplex structures. Enzymic methylation of Arg by protein arginine *N*-methyltransferase (PRMT) 3 reduced the binding RGG domain of EWS to G-quadruplexes but increased its binding to single-strand DNA and RNA. PRMT1 (a homologue of PRMT3) reduced transcriptional activity of EWS through nuclear exclusion of EWS by methylation of arginine residues [62]. It suggests that the regulation of nucleic acids structure specific binding by EWS might play an important role in regulating the transcriptional activity. Substitution of the phenylalanines in the RGG domain in EWS eliminated almost completely G-quadruplex bindings. These findings indicate that the phenylalanines and guanidinium groups of the arginines in RGG domain of EWS are important for binding of EWS to the G-quadruplex. Moreover, not only Arg-Gly-Gly repeats sequences but also Arg and Pro rich sequence in RGG domain of C-terminus are important for its specific binding to G-quadruplex. The RGG domain has been widely observed at divergent kinds of RNA-binding proteins [63,64]. These data might contribute to understanding of the nucleic acids binding specificity of RNA-binding proteins containing RGG domains.

## Conclusions

Folding of RNA into divergent structures is constrained by specific RNA sequences under physiological conditions. Recent analyses revealed that several ncRNAs form specific conformations in sequence-and structure-specific manners, mimicking an open promoter structures and G-quadruplex structures. In living cells, specific three-dimensional structures of these ncRNAs could contribute to regulation of transcription. It remains however unclear whether a role of EWS in transcription is fixed by its ability to target a specific RNA structure. Janknecht et al. reported that overexpression of EWS in RK13 and AKR cells leads to activation of the c-fos, Xvent-2, and ErbB2 promoters, while EWS did not bind to double-stranded DNAs in these promoters [65]. These data suggest that EWS might function as a transcriptional regulator upon its binding with structure-specific RNAs. On the other hand, Kingston and Dejardin purified the human telomeric chromatins using proteomics technology with isolated chromatin segments and, found that TLS binds to telomeres [43]. Telomeres are transcribed form the telomeric C-rich strand, giving rise to TERRA r(UUAGGG)_4 _which forms G-quadruplex *in vivo*. TLS might bind to TERRA and regulate transcription of TERRA.

## List of abbreviations

ncRNAs: noncoding RNAs; TLS: translocated in liposarcoma; EWS: Transcriptional factor Ewing's sarcoma; HAT: histone acetyltransferase; TERRA: telomeric repeat containing RNA.

## Competing interests

The authors declare that they have no competing interests.

## Authors' contributions

TO and RK made substantial contributions to conception, design and been involved in drafting the manuscript. RK has given final approval of the version to be published. Each author has participated sufficiently in the work to take public responsibility for appropriate portions of the content. TO and RK read and approved the final manuscript.
